# A European phylogeography of *Rhinanthus minor* compared to *Rhinanthus angustifolius*: unexpected splits and signs of hybridization

**DOI:** 10.1002/ece3.276

**Published:** 2012-07

**Authors:** Jérôme Vrancken, Christian Brochmann, Renate A Wesselingh

**Affiliations:** 1 Biodiversity Research Centre, Earth & Life Institute, Université catholique de LouvainCroix du Sud 4–5, B-1348 Louvain-la-Neuve, Belgium; 2 National Centre for Biosystematics, Natural History Museum, University of OsloP.O. Box 1172 Blindern, NO-0318 Oslo, Norway

**Keywords:** AFLP, cpDNA, hybridization, introgression, phylogeography, *Rhinanthus*

## Abstract

*Rhinanthus minor* and *Rhinanthus angustifolius* (Orobanchaceae) are annual hemiparasites, which occur sympatrically in Europe and are known to hybridize. We studied chloroplast and nuclear (amplified fragment length polymorphism [AFLP]) diversity in *R. minor* and compared genetic structuring in this species with *R. angustifolius* by analyzing the AFLP data for both species simultaneously. The AFLP data revealed that populations in Italy, Greece, and southeast Russia initially identified as *R. minor* were so distant from the other *R. minor* populations that they probably belong to another, yet unidentified taxon, and we refer to them as *Rhinanthus* sp. *R. minor* s.s. showed a clear geographic genetic structure in both the chloroplast DNA (cpDNA) and nuclear genome. The simultaneous analysis of both species shed new light on the previously published findings for *R. angustifolius*, because some populations now turned out to belong to *R. minor*. The admixture analysis revealed very few individuals of mixed *R. minor–R.angustifolius* ancestry in the natural populations in the west of Europe, while admixture levels were higher in the east. The combined haplotype network showed that haplotype H1 was shared among all species and is likely to be ancestral. H2 was more abundant in *R. angustifolius* and H3 in *R. minor*, and the latter probably arose from H1 in this species in the east of Europe. The occurrence of H3 in *R. angustifolius* may be explained by introgression from *R. minor*, but without interspecific admixture, these are likely to have been old hybridization events. Our study underlines the importance of including related species in phylogeographic studies.

## Introduction

A growing number of studies have demonstrated wide-ranging sharing of chloroplast DNA (cpDNA) haplotypes across species boundaries in plants, often trees (e.g., *Quercus*: Petit et al. 2002; *Betula*: Palmé et al. 2003; *Fraxinus*: Heuertz et al. 2006). The presence of an identical cpDNA haplotype in different species may result from three basic processes: convergent evolution, incomplete lineage sorting of ancient polymorphisms, and interspecific gene flow. Plants have for long been known for their high levels of interspecific gene flow (Rieseberg and Soltis 1991), and many studies addressing cpDNA haplotype sharing have indeed concluded that hybridization and subsequent backcrosses to one of the parental species account for this pattern (Gutiérrez Larena et al. 2002; Paun et al. 2006; Eidesen 2007; Lihová et al. 2007).

Such haplotype sharing resulting from hybridization is an important issue in phylogeographic studies as it may compromise the interpretation of species demographic histories by superimposing effects of recent processes onto older ones. Exploring and quantifying gene flow between species is thus of prime importance in plant phylogeography.

**Figure 1:**
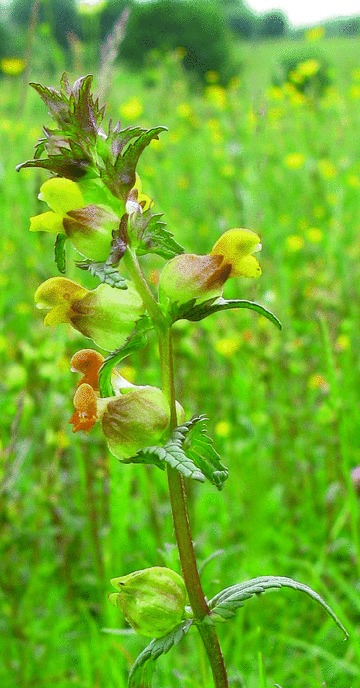
Inflorescence of *Rhinanthus minor* L., photographed by R. A. Wesselingh in population be1 (Braine-le-Château, Belgium), 27 May 2008.

The genus *Rhinanthus* L. (Orobanchaceae) offers a good model system for the study of genetic interactions between closely related species. The genus comprises 25 annual hemiparasitic species in Europe (von Soó and Webb 1972), and hybridization is frequent (Campion-Bourget 1980). *Rhinanthus angustifolius* C. C. Gmelin and *Rhinanthus minor* L. (Fig. 1) are the two most widespread species of the genus and co-occur in a large part of their ranges. Both are grassland species with overlapping ecological requirements (Ducarme and Wesselingh 2010). They are diploid (2*n* = 22, Hambler 1954) and self-compatible, and their yellow flowers are pollinated by bumblebees (Kwak 1980; Natalis and Wesselingh 2012). The heavy seeds do not disperse very far and have a short life span, so no persistent seed bank is formed (ter Borg 1972). They have been known for a long time to form fertile hybrids (Chabert 1899; Kwak 1978; Campion-Bourget 1980). In natural, mixed populations, most hybrids are genetically close to *R. angustifolius* (Kwak 1980; Ducarme et al. 2010), probably due to a combination of the higher selfing rate of *R. minor* (Ducarme 2008) and a preference of the visiting bumblebees for *R. angustifolius* (Natalis and Wesselingh 2012). This should facilitate introgression from *R. minor* to *R. angustifolius*, and since F_1_ hybrids predominantly have the *R. minor* cytoplasm, due to a low germination rate of F_1_ seeds formed on *R. angustifolius* (Kwak 1979), this could lead to cpDNA introgression in the same direction as well.

In a previous study of the phylogeography of *R. angustifolius* (Vrancken et al. 2009), we observed different patterns for cpDNA and nuclear diversity. The nuclear variation, as inferred from amplified fragment length polymorphism (AFLP) markers, was well-structured geographically, while no clear pattern could be observed in the cpDNA sequence diversity. As the most likely explanation for the absence of geographic structure within this usually phylogeographically informative marker system, we suggested cpDNA introgression through hybridization with *R. minor*.

In the present study, we investigate the level and geographic structuring of cpDNA and nuclear variation in 57 populations of *R. minor* sampled over most of its range, based on sequencing of two noncoding cpDNA regions and AFLP. Additionally, we included known natural hybrids between *R. minor* and *R. angustifolius* and a subset of the previously analyzed populations of *R. angustifolius* in the AFLP analysis to characterize the hybrids genetically and facilitate direct comparison between the species. These results were used to compare the patterns observed in *R. minor* with those previously obtained for *R. angustifolius*, to examine whether hybridization and cpDNA introgression rather than incomplete lineage sorting or convergent evolution could explain the absence of cpDNA structure in *R. angustifolius*.

## Materials and Methods

### Sampling and DNA isolation

**Figure 2:**
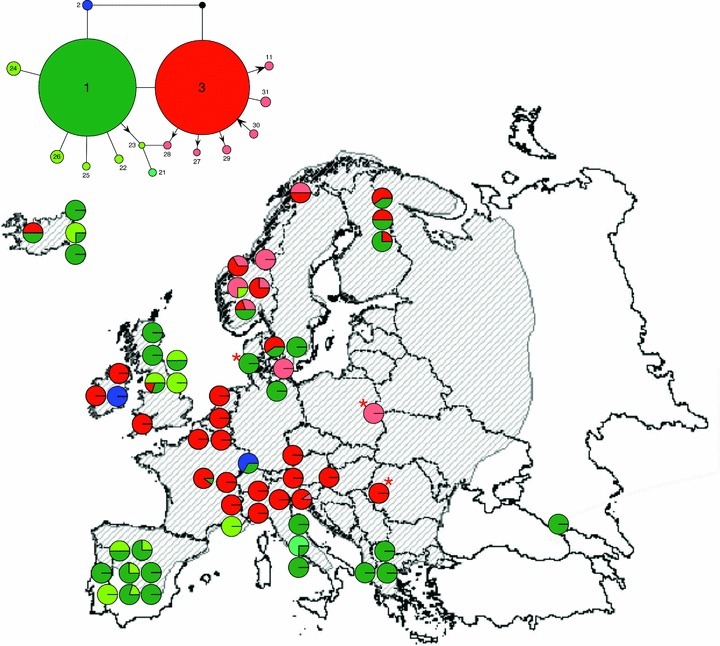
Parsimony network connecting the 14 cpDNA haplotypes detected in *Rhinanthus minor* s.l. and the geographic distribution of the cpDNA haplotypes. Shaded areas represent the distribution range of *Rhinanthus minor* in Europe (redrawn from Hultén and Fries 1986). In the parsimony network, the size of the circles is proportional to the haplotype frequency. Arrows indicate indels, pointing toward the shorter of the two sequences, with the size of the arrowhead proportional to the size of the indel. The red asterisks indicate three populations analyzed as *Rhinanthus angustifolius* in Vrancken et al. (2009), but identified as *R. minor* in this study.

Fifty-seven natural populations of *R. minor* were sampled from most of its distribution range in Europe (Fig. 2; Table 1). Seeds from four to 10 individuals at a minimum interindividual distance of 10 m were sampled from each population. DNA was isolated from a single seed using the Invisorb Spin Plant Mini Kit (Stratec Molecular, Berlin) and diluted with 50 µL of elution buffer. Seeds from populations that we did not sample ourselves were sown and the plants cultivated in a greenhouse in order to verify species identity based on morphology.

**Table 1:** Sampling locations, chloroplast DNA (cpDNA) haplotypes, Nei's diversity for the cpDNA data, amplified fragment length polymorphism (AFLP) groups, Nei's unbiased gene diversity for AFLP, and rarity index (DW) of AFLP markers for the 57 populations of *Rhinanthus minor* s.l., population codes followed by an * are *Rhinanthus* sp. populations. The number of individuals analyzed is given for each marker. Values in parentheses are percentages of cpDNA haplotypes

Population	Country	Latitude (°N)	Longitude (°E)	*n* cpDNA	cpDNA haplotypes	cpDNA diversity	*n* AFLP	AFLP diversity	DW
at1	Austria	47°45′	13°03′	4	H3	0.000	4	0.009	0.155
at2	Austria	47°11′	13°07′	10	H3(90) H27(10)	0.200	9	0.090	0.221
at3	Austria	47°15′	13°34′	4	H3	0.000	4	0.017	0.228
be1	Belgium	50°40′	04°14′	4	H3	0.000	5	0.007	0.219
be2	Belgium	50°11′	05°12′	10	H3	0.000	10	0.040	0.155
dk1	Denmark	54°47′	11°53′	2	H1	0.000	0	—	—
en1	England	54°37′	-00°39′	4	H1(50) H24(50)	0.670	4	0.048	0.140
en2	England	54°10′	-01°06′	10	H1(30) H3(20) H24(50)	0.690	10	0.075	0.163
en3	England	54°18′	-00°49′	4	H24	0.000	4	0.026	0.157
fi1	Finland	66°22′	29°32′	4	H1(75) H3(25)	0.500	4	0.043	0.144
fi2	Finland	66°22′	29°35′	10	H1(40) H3(60)	0.500	10	0.106	0.217
fi3	Finland	66°22′	29°35′	4	H1(50) H3(50)	0.510	4	0.100	0.201
fr1	France	45°54′	06°34′	4	H3	0.000	4	0.032	0.312
fr2	France	44°50′	06°20′	4	H22	0.000	4	0.107	0.166
fr3	France	47°20′	03°36′	10	H1(10) H3(90)	0.200	10	0.117	0.301
fr4	France	45°14′	06°11′	4	H3	0.000	4	0.009	0.183
de1	Germany	48°00′	07°52′	3	H1(33) H2(67)	0.670	3	0.033	0.141
gr1*	Greece	41°07′	22°20′	10	H1	0.000	10	0.031	0.713
gr2*	Greece	40°50′	21°59′	4	H1	0.000	4	0.007	0.676
gr3*	Greece	40°37′	22°06′	4	H1	0.000	4	0.015	0.518
hu1	Hungary	47°28′	17°02′	3	H3	0.000	3	0.078	0.142
is1	Iceland	65°19′	-13°50′	4	H1	0.000	3	0.065	0.187
is2	Iceland	65°35′	-14°04′	4	H1	0.000	4	0.022	0.149
is3	Iceland	65°37′	-14°16′	4	H1(25) H25(75)	0.500	4	0.031	0.141
is4	Iceland	65°39′	-20°16′	4	H1(50) H3(50)	0.670	4	0.040	0.164
ir1	Ireland	53°07′	-09°17′	4	H3	0.000	4	0.045	0.208
ir2	Ireland	53°20′	-07°30′	4	H3	0.000	4	0.015	0.267
ir3	Ireland	54°07′	-07°35′	4	H2	0.000	4	0.018	0.149
it1*	Italy	41°50′	13°20′	4	H1	0.000	4	0.042	0.508
it2*	Italy	42°46′	13°37′	4	H1(25) H21(75)	0.500	4	0.156	1.894
it3*	Italy	42°47′	13°36′	10	H1	0.000	9	0.031	0.628
nl1	Netherlands	52°17′	05°44′	4	H3	0.000	4	0.045	0.137
nl2	Netherlands	53°18′	05°04′	4	H3	0.000	4	0.039	0.136
no1	Norway	61°23′	08°18′	3	H3(67) H27(33)	0.670	3	0.143	0.378
no2	Norway	61°35′	08°55′	10	H1(50) H3(20) H29(30)	0.690	10	0.052	0.123
no3	Norway	61°35′	09°00′	4	H23(25) H28(75)	0.500	4	0.010	0.179
no4	Norway	61°29′	08°37′	4	H3(75) H29(25)	0.500	4	0.050	0.155
pt1	Portugal	40°12′	-08°25′	4	H1	0.000	4	0.136	0.227
ru1*	Russia	43°27′	41°42′	10	H1	0.000	10	0.047	1.013
sc1	Scotland	56°09′	-03°20′	4	H1	0.000	4	0.049	0.131
sc2	Scotland	56°09′	-03°21′	4	H1	0.000	4	0.007	0.129
es1	Spain	40°59′	-05°91′	4	H1(50) H26(50)	0.670	4	0.044	0.150
es2	Spain	40°27′	-05°65′	4	H26	0.000	4	0.011	0.172
es3	Spain	40°32′	-05°44′	4	H1	0.000	4	0.029	0.176
es4	Spain	40°31′	-05°42′	4	H1(75) H26(25)	0.500	4	0.040	0.200
es5	Spain	40°34′	-05°17′	4	H1	0.000	4	0.031	0.164
es6	Spain	40°28′	-05°14′	10	H1(80) H26(20)	0.360	10	0.031	0.164
es7	Spain	40°36′	-05°14′	4	H1(75) H26(25)	0.500	4	0.027	0.150
se1	Sweden	56°45′	13°00′	4	H1	0.000	4	0.025	0.129
se2	Sweden	56°40′	12°48′	10	H1(40) H3(60)	0.530	10	0.055	0.185
se3	Sweden	63°17′	12°20′	4	H31	0.000	4	0.018	0.164
se4	Sweden	68°21′	18°49′	4	H3(50) H31(50)	0.670	7	0.033	0.122
se5	Sweden	55°20′	13°10′	4	H30	0.000	4	0.055	0.123
ch1	Switzerland	46°27′	09°35′	4	H3	0.000	4	0.003	0.210
ch2	Switzerland	46°27′	09°33′	10	H3	0.000	10	0.037	0.146
ch3	Switzerland	46°26′	09°47′	4	H3	0.000	4	0.031	0.174
cy1	Wales	51°39′	-04°43′	4	H3	0.000	3	0.045	0.166

For *R. angustifolius*, we randomly selected a subset of up to four individuals (Appendix A1) from most of the populations analyzed in the previous paper (Vrancken et al. 2009). AFLPs for these plants were rerun in the laboratory and reanalyzed together with *R. minor* to allow direct comparison between the two species.

To examine whether interspecific hybrids could be detected in the natural populations, we used plants of known descent as reference. In a garden experiment conducted in 2007, a mixed population was created with potted plants grown from seed from a *R. minor* population in the nature reserve Housta-Dardenne (Braine-le-Château, Belgium; population be1, Table 1) and from a *R. angustifolius* population in the reserve Doode Bemde (Oud-Heverlee, Belgium; population BE3; Appendix A1). Seeds were obtained by open pollination, and the potential F_1_ hybrid status of these seeds was assessed by DNA extraction from a single seed and the use of one microsatellite and two species-specific random amplified polymorphic DNA (RAPD) markers (Ducarme 2008; Ducarme et al. 2008). We used the DNA of four of the identified F_1_ hybrids, and DNA from leaf samples of their respective maternal parents, two *R. angustifolius* and two *R. minor* plants, in the AFLP analysis. We also used DNA extracted from leaf samples collected in 2004 in a naturally mixed population of the two species in the nature reserve of Kalkense Meersen, Uitbergen (province of Oost-Vlaanderen, Belgium), where the species have grown together for at least 12 years (Ducarme et al. 2010). The hybrid status of these plants was assessed with eight species-specific markers, five RAPD, and three inter-simple sequence repeat (ISSR) markers (Ducarme and Wesselingh 2005; Ducarme et al. 2010), resulting in a hybrid index that ranged from –4 (*R. minor*) to +4 (*R. angustifolius*). We used DNA of nine plants, two plants with index +4, two with +2, two with 0 and all markers present (and therefore potential F_1_ hybrids), two with –2, and one with –4.

### cpDNA sequencing

We have shown that cpDNA is inherited maternally in *R. angustifolius* (Vrancken and Wesselingh 2010). The non-coding *trn*T*trn*L spacer and the *trn*L intron were amplified with primers a-b and c-d, respectively (Taberlet et al. 1991). Fragments were amplified by polymerase chain reaction (PCR) in a 25 µL volume using 3 µL extracted DNA, 0.025 U/µL Taq DNA polymerase (Roche Applied Science, Penzberg, Germany), 2.5 µL PCR buffer 10×, 1.5 mM MgCl_2_, 0.2 mg/mL bovine serum albumin, 0.2 µM dNTPs, and 0.25 µL of each 20 µM primer. Amplification was performed for 5 min at 94°C (1×), 1 min at 94°C, 1 min at 55°C, 2 min at 72°C (30 cycles), and 7 min at 72°C (1×) using the Gene Amp PCR System 9700 thermocycler (Perkin-Elmer, Waltham, Massachusetts, USA). The amplification products were checked on 1% agarose gels and samples were diluted 10 times before sequencing. Sequencing reactions were performed forward using 1 µL of 5 µM a or c primers and 4 µL of diluted PCR product. PCR products were dye-labeled using a Big Dye Terminator Kit (Applied Biosystems, Foster City, California, USA) and reactions were run on an ABI 3100 automated sequencer (Applied Biosystems, Foster City, California, USA). Sequences were manually aligned using BioEdit (Hall 1999).

### AFLP fingerprinting

The three primer combinations used for AFLP analysis of *R. minor* were identical to those used for *R. angustifolius* in our previous study (Vrancken et al. 2009). The AFLP protocol followed Gaudeul et al. (2000). Amplification products for the three primer combinations were mixed in the following proportions: 2 µL—FAM, 2 µL—NED, and 3 µL—VIC and diluted with 14 µL purified water. A total of 3 µL of this mix were loaded with 11.7 µL HiDi formamide and 0.3 µL GENESCAN ROX 500 on a PCR plate and run on an ABI 3100. ABI chromatographs were imported and analyzed in the GENESCAN 3.7 analysis software (Applied Biosystems, Foster City, California, USA). AFLP fragments were scored with GENOGRAPHER 1.6 (http://hordeum.oscs.montana.edu/genographer/). The AFLP reactions and analyses of one randomly chosen individual for each of 24 *R. minor* populations (6% of the 399 individuals analyzed) were duplicated to serve as a blind sample in order to test the reproducibility of the scoring and to calculate the error rate. Two other *R. minor* individuals were run on each plate to check for among-plate repeatability (Bonin et al. 2004).

### cpDNA data analysis

Analyses were performed on the *R. minor* dataset consisting of the combined cpDNA regions. Indels were treated as a single mutation event. An unrooted haplotype network also including the haplotypes found in *R. angustifolius* (Vrancken et al. 2009) was constructed using the program TCS version 1.21 (Clement et al. 2000). Diversity and population differentiation for *R. minor* only were calculated with the program PERMUT version 2.0 (http://www.pierroton.inra.fr/genetics/labo/Software/Permut/; Pons and Petit 1996). Computed parameters included the mean within-population gene diversity (*h*_S_), the total gene diversity (*h*_T_), the coefficient of genetic differentiation over all populations (*G*_ST_), and equivalent parameters computed by taking into account the similarities between haplotypes (*v*_S_, *v*_T_, *N*_ST_). In order to statistically compare the two parameters of genetic differentiation (Burban et al. 1999), a permutation test with 1000 random permutations of haplotype identities was performed using the same program. Additionally, Nei's gene diversity was computed for all populations with ARLEQUIN version 3.0 (Excoffier et al. 2005). Isolation by distance was assessed with a Mantel test as described below for the AFLP data, with molecular differentiation between-population pairs quantified with *F*_ST_ using the results of an analysis of molecular variance (AMOVA in ARLEQUIN 3.0).

### AFLP data analysis

For the entire dataset (including *R. minor*, *R. angustifolius* and known hybrids), we constructed a binary matrix by scoring bands with sizes between 50 and 500 base pairs (bp). Presence of unambiguous bands was scored as 1 and absence as 0. Bands that were not perfectly reproducible between replicates were eliminated from the matrix (23 out of 204 bands). We calculated a matrix of genetic distances between individuals in the full dataset using the module Restdist in PHYLIP 3.69 (Felsenstein 2004), and a Neighbor-Joining (NJ) tree was then produced with PHYLIP 3.69 and drawn with FIGTREE v.1.3.1 (Andrew Rambaut, http://tree.bio.ed.ac.uk/).

Genetic structuring was further investigated using a Bayesian model-based clustering algorithm implemented in STRUCTURE (Pritchard et al. 2000). We analyzed our dataset with STRUCTURE version 2.3.2.1 at the Bioportal, University of Oslo (http//www.bioportal.uio.no) using the admixture model, uncorrelated allele frequencies models, no prior information, and the following parameters: *K* from 1 to 12, 25 replicate runs for each *K*, a burn-in period of 2 × 10^5^ and 10^6^ iterations. The optimal number of clusters *K* in our dataset was selected using the methods described by Evanno et al. (2005) using the R script Structure-sum-2011 (Ehrich 2006). *F*_ST_ computations for *R. minor* populations were conducted using ARLEQUIN version 3.0 (Excoffier et al. 2005). Pairwise *F*_ST_ values for the STRUCTURE clusters were computed with AFLPSURV 1.0 (Vekemans et al. 2002) by pooling populations of all species in each cluster. Gene diversity within *R. minor* populations and STRUCTURE clusters (entire dataset), estimated by the Nei's unbiased diversity estimator were computed using AFLPSURV 1.0 (Vekemans et al. 2002). The R script AFLPdat (Ehrich 2006) was used to calculate the frequency-down-weighted (DW) marker value for *R. minor* populations. This DW value is an estimator of differentiation expressed by the rarity of markers present in a population (Schönswetter and Tribsch 2005). To test for isolation by distance for *R. minor* populations, the correlation between pairwise *F*_ST_ values computed with ARLEQUIN and geographical distances between the populations in kilometers between the populations was estimated with a Mantel test (1000 replications) using the Microsoft Excel add-on POPTOOLS (http://www.poptools.org/).

## Results

### cpDNA: molecular variation, genetic diversity, and geographic structure in *R. minor*

The alignment of the concatenated matrix of the *trn*T-*trn*L spacer and *trn*L intron sequences for the 295 successfully sequenced individuals (Table 1) was 1130 bp long. Twelve variable sites were found (1.1%): eight substitutions and four indels. These mutations distinguished 14 haplotypes shown on the parsimony network (Fig. 2). Two haplotypes were frequent: H1 and H3 were found in 50 out of the 57 populations. Together they were present in 81% of the individuals, while the other haplotypes were rare and often restricted to one population (Table 2).

**Table 2:** Sequence polymorphism in the 14 chloroplast DNA (cpDNA) haplotypes found in *Rhinanthus minor*. Haplotype identification numbers were given according the numbering started for *Rhinanthus angustifolius* haplotypes in Vrancken et al. (2009). The numbers above each column indicate the nucleotide positions, and Freq is the haplotype frequency across all populations

	*trn*T*-trn*L spacer										*trn*L intron			
				
	94	254	273	279	289	313	324	386	506	621	294	361	Freq	Present in population(s)
H1	TATAGTA	A	AATATTA	AAATATT	A	T	A	**- - - - - - - - - - - - - - - - - - -**	G	A	C	A	0.41	dk1, en1-2, fi1-3, fr3, de1, gr1-3, is1-4, it1-3, no2, pt1, ru1, sc1-2, es1, es3-7, se1-2
H2	………	.	………	………	.	.	C	…………………	.	.	.	.	0.02	de1, ir2
H3	………	.	………	………	.	.	.	…………………	.	.	T	.	0.40	at1-3, be1-2, en2, fi1-3, fr1, fr3-4, hu1, is4, ir1-2, nl1-2, no1-2, no4, se2, ch1-3, cy1
H21	………	.	………	- - - - - - -	.	.	.	…………………	C	.	.	.	0.01	it2
H22	………	.	………	………	.	G	.	…………………	.	.	.	.	0.01	fr2
H23	………	.	………	- - - - - - -	.	.	.	…………………	.	.	.	.	0.00	no3
H24	………	C	………	………	.	.	.	…………………	.	.	.	.	0.04	en1-3
H25	………	.	………	………	T	.	.	…………………	.	.	.	.	0.01	is3
H26	………	.	………	………	.	.	.	…………………	.	C	.	.	0.03	es1-2, es4, es6-7
H27	**- - - - - - -**	.	………	………	.	.	.	…………………	.	.	T	.	0.01	at2, no1
H28	………	.	………	- - - - - - -	.	.	.	…………………	.	.	T	.	0.01	no3
H29	………	.	**- - - - - - -**	………	.	.	.	…………………	.	.	T	.	0.01	no2, no4
H30	………	.	………	………	.	.	.	TCTATCCTATATATTTATT	.	.	T	.	0.01	se5
H31	………	.	………	………	.	.	.	…………………	.	.	T	C	0.02	se3-4

We defined five informal haplotype groups: H1, close to H1, H3, close to H3, and H2 (Fig. 2). Among the 57 populations, 36 were monomorphic for one haplotype and 11 had two close haplotypes, H1 and close to H1, or H3 and close to H3. Combinations of haplotypes H1, H2, and H3 or derived haplotypes were observed in 10 populations, mainly in Scandinavia, and two of these had a total of three haplotypes detected among 10 sampled individuals. The haplotype groups had different geographical distributions (Table 2; Fig. 2). Haplotype H3 was more abundant in the alpine region and in the central part of our sampling area, and the haplotypes derived from it were almost exclusively found in Scandinavia. H1 was found in all the southern regions and was predominant in the Atlantic and northwestern Europe, while its derived haplotypes were more widespread than for H3, but each was only found in a single population except for H26, which occurred in three Spanish populations. The mean within-population gene diversity for western populations was 0.21, with values ranging from 0 to 0.69 (Table 1). We observed high genetic differentiation (*G*_ST_ = 0.69) among the western and northern populations, excluding the southeastern populations in Italy, Greece, and Russia. Differentiation was significantly higher when similarity between haplotypes was taken into account (*N*_ST_ = 0.77). The Mantel test was significant for western populations (test statistic = 0.100, *P* = 0.002), showing that geographically distant populations were more differentiated than adjacent ones. As only two haplo-types were observed in the southeastern populations (H1 and H21), gene diversity within populations was low (mean: 0.07) and tests for differentiation and isolation by distance were not performed.

### AFLP variation in *R. minor*

We scored 181 polymorphic markers in the 399 individuals that were successfully analyzed: 289 that were originally identified as *R. minor* (Table 1), 93 belonging to *R. angustifolius*, and 17 individuals used in the hybrid detection test. The average reproducibility was 97.5% for the three primer pairs.

**Figure 3:**
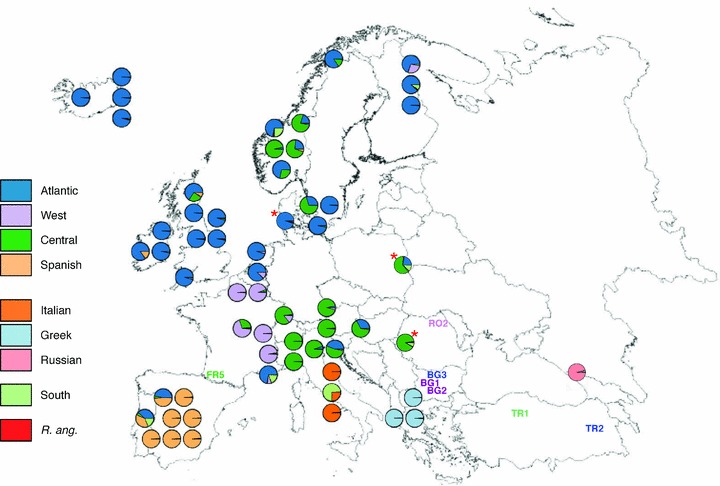
Geographic distribution of the nine clusters detected in the STRUCTURE analysis of the AFLP data. The pie charts depict population means of *q*-values for each cluster calculated from individual averages for the three highest scoring STRUCTURE runs for *K* = 9 for populations of *Rhinanthus minor* s.l., while the uppercase population codes indicate the locations of the *Rhinanthus angustifolius* populations discussed in the text.

In the combined dataset including both *R. minor* and *R. angustifolius* samples, the STRUCTURE analyses identified nine clusters as being most optimal (Appendix A2). The three runs with the highest LnP(D) values, over –19,850, all had the same composition, with standard deviations for *q*-values of individual samples of 0.015 or less. Seven of the clusters contained predominantly *R. minor* individuals, while most of the *R. angustifolius* individuals were assigned to a single cluster. The remaining cluster, South, contained three individuals from an Italian population, all four plants from the G2 group (a single population) previously detected in *R. angustifolius* (Vrancken et al. 2009), plus some isolated individuals from various other *R. minor* populations. The seven *R. minor* clusters had different geographical distributions (Fig. 3). The Spanish, Italian, Greek, and Russian clusters were restricted almost completely to their respective countries in the south of Europe, while the other clusters had much wider distributions. The Atlantic cluster was strongly represented in Iceland, the British Isles, and northern Scandinavia, while the Central cluster was dominant in the Alps and also represented in western Scandinavia. The West cluster was only found in France, Belgium, and the Netherlands, and in one Finnish population.

**Figure 4:**
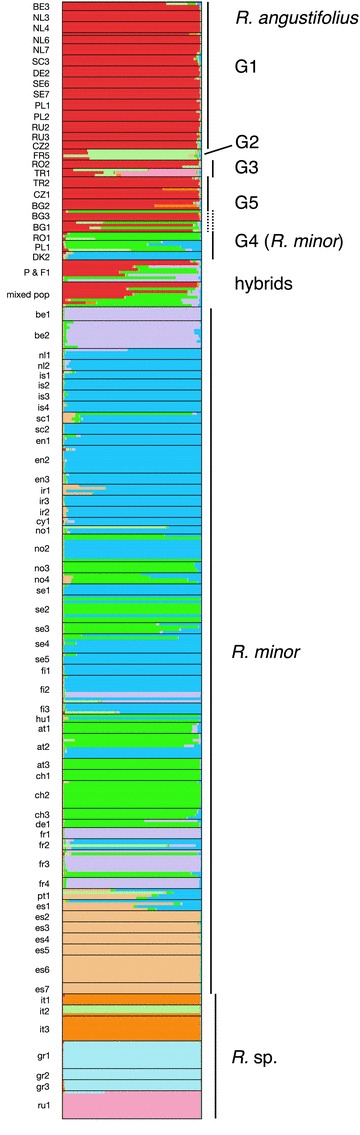
Within-individual proportions of ancestry (*q*-values) produced by the STRUCTURE analysis. The colors used for the nine clusters are the same as in Fig. 3; the population codes can be found in Table 1 and Appendix A1. From top to bottom: *Rhinanthus angustifolius* populations (uppercase population codes), with the AFLP groups from Vrancken et al. (2009) indicated on the right; P and F1: four F_1_ hybrids surrounded by their maternal parents; mixed pop: the mixed population in descending order of hybrid index (from +4 to –4); the *R. minor* and *Rhinanthus* sp. populations (lowercase population codes).

Our previous study on *R. angustifolius* (Vrancken et al. 2009) distinguished five AFLP groups for this species. The reanalysis of part of the samples in combination with *R. minor* shed new light on one of these groups, G4. The Danish population DK2, which was geographically isolated from the other, eastern European populations in this group, fell squarely into the *R. minor* Central cluster (Fig. 4) and turned out to be genetically closest to *R. minor* samples from Iceland. We had not been able to grow plants from the seeds of this Danish population (they were too old to germinate), and an inquiry at the source of the seeds, the Botanical Garden of the University of Copenhagen, could not confirm the identity of the plants in the wild population where they had been sampled, but based on our results, it is highly likely that the seeds had been sampled on *R. minor* plants. The remaining populations in the G4 group, from Poland (PL1) and Romania (RO1), belonged to the *R. minor* Central cluster (Fig. 4), and again, these should be considered as *R. minor* and not *R. angustifolius*. The two Bulgarian populations BG1 and BG2, which had been classified in two AFLP groups by Vrancken et al. (2009), G4 and G5, turned out to contain both *R. angustifolius* and *R. minor* plants, 2 + 2 in BG1 and 3 + 1 in BG3.

**Figure 5:**
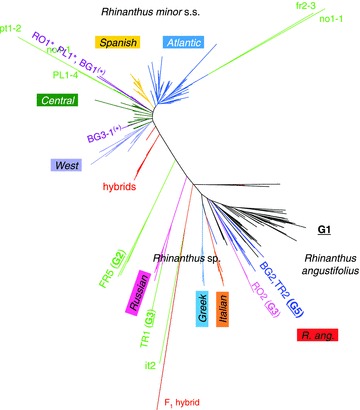
Unrooted Neighbor-Joining (NJ) tree based on AFLP data for *Rhinanthus minor*, *Rhinanthus* sp., and *R. angustifolius*. The names of eight of the nine AFLP clusters detected in the STRUCTURE analysis presented in this paper are shown in colored boxes. Branches belonging to the ninth cluster, South, are indicated in light green. AFLP groups (G1–G5) from Vrancken et al. (2009) are underlined and bold, other labels indicate hybrids (red) and specific populations or individuals discussed in the text, population codes as in Fig. 4, Table 1, and Appendix A1.

The NJ tree (Fig. 5) showed a clear separation among plants identified as *R. minor*. The main bulk of *R. minor* plants, belonging to the Atlantic, West, Central, and Spanish clusters, was found at one end, while the remaining clusters (Italian, Greek, and Russian) are at the opposite end, close to the *R. angustifolius* AFLP cluster. The South cluster did not form a single branch; its members were spread out over the tree, often on very long branches (Fig. 5).

Pairwise *F*_ST_ computed for the clusters (Table 3) confirmed the strong differentiation between the three southeastern groups (Italian, Greek, and Russian) on the one hand and the remaining *R. minor* clusters on the other hand. These southeastern *R. minor* clusters were less differentiated from *R. angustifolius* than from the other *R. minor* clusters.

**Table 3:** Pairwise *F*_ST_ values between AFLP clusters detected by STRUCTURE (under diagonal) and Nei's unbiased gene diversity within each AFLP group (bold on diagonal)

	*n*	Atlantic	West	Central	Spanish	Italian	Greek	Russian	South	*R. angustifolius*
Atlantic	112	**0.1122**								
West	35	0.2021	**0.1530**							
Central	79	0.1155	0.1225	**0.1280**						
Spanish	35	0.2289	0.2981	0.2178	**0.1067**					
Italian	14	0.5492	0.4695	0.5188	0.5650	**0.1444**				
Greek	18	0.5738	0.5026	0.5397	0.5918	0.4120	**0.1457**			
Russian	12	0.4903	0.4229	0.4553	0.5065	0.4136	0.4351	**0.1915**		
South	13	0.3282	0.2748	0.2993	0.3783	0.3269	0.3931	0.2976	**0.2205**	
*R. angustifolius*	81	0.4449	0.3783	0.4115	0.4828	0.2862	0.3680	0.3583	0.1968	**0.1614**

Based on these clear differences observed between the northwestern and southeastern clusters, populations from these two areas were treated separately in subsequent analyses and will be referred to as “*R. minor”* for individuals of the Atlantic, West, Central, and Spanish clusters and “*Rhinanthus* sp.” for plants from Italy, Greece, and Russia.

On average, gene diversity within *R. minor* populations was 0.045, while it was 0.047 for the *Rhinanthus* sp. populations. High values were observed in the populations where admixture between AFLP clusters was observed (Table 1). The rarity of AFLP markers within populations estimated by the DW values in *R. minor* ranged from 0.137 in south Sweden to 0.631 in Norway (Table 1), with an average of 0.241. The slope of the regression of DW values on latitude was positive, but not significant (*P* = 0.423). Among the southeastern *Rhinanthus* sp. populations, DW values were much higher, on average 0.850, with the highest score recorded in Russia (1.89). Genetic differentiation among Atlantic, West, Central, and Spanish *R. minor* populations was relatively high (*F*_ST_ = 0.581), and the Mantel test (test statistic = 0.21, *P* < 0.001) showed an effect of isolation by distance. For the southeastern *Rhinanthus* sp. populations genetic differentiation was much stronger (*F*_ST_ = 0.835) and the Mantel test (test statistic = 0.77, *P* < 0.001) demonstrated a strong effect of isolation by distance.

### AFLP structuring in *R. angustifolius*

Of the five AFLP groups presented in Vrancken et al. (2009), group G4 turned out to belong entirely to *R. minor*. Group G1, the largest group, formed part of the *R. angustifolius* AFLP cluster in the current analysis, together with group G5 and one population in G3—the Romanian population RO2. The other population in G3, the Turkish population TR1, showed a mixture of the Russian, the *R. angustifolius*, and the South cluster in the current STRUCTURE analysis (Fig. 4), and it is on the Russian branch of the NJ tree (Fig. 5), which may indicate a mixed *Rhinanthus* sp.–*R. angustifolius* ancestry. The French population FR5, which formed a distinct AFLP group on its own, G2, in the previous analysis, now mainly grouped with the South cluster. Its intermediate position on the NJ tree (Fig. 5) might indicate a mixed ancestry between *R. minor* and *R. angustifolius*, but since the South cluster is not confined to a single position on the NJ tree, this is harder to interpret, and a third *Rhinanthus* species may even be involved.

### Admixture: known hybrids

The F_1_ hybrids from the garden experiment had *q*-values for the *R. angustifolius* cluster close to the expected value of 50%: 50% and 54% for the two F_1_ hybrids produced by *R. angustifolius* mothers, and 40% and 45% for the two hybrids produced by *R. minor* mothers (Fig. 5). The two intermediate hybrids with hybrid index 0 from the mixed natural population had comparable scores, 50% and 44%. More deviation from the expected values was found for the hybrids with hybrid indices closer to either of the parental species: the two hybrids with hybrid index +2 for eight RAPD/ISSR markers, which were expected to have *q*-values around 75%, had scores of 46% and 90%, while the two hybrids with index –2 (expected *q*-value 25%) had 2% and 16%. The plants with +4 (97% and 97%) and –4 (0.2%) were probably indeed pure *R. angustifolius* and *R. minor*, respectively, without signs of hybridization.

### Admixture in *R. minor* and *Rhinanthus* sp

Within *R. minor*, 57 individuals had a maximum *q*-value of less than 0.95, corresponding to 23% of the 245 individuals in the four main clusters. For the individuals in the Atlantic cluster, most of the admixture was with West and Central, but some populations in Scotland and Ireland contained plants with high *q*-values for the Spanish cluster, between 0.06 and 0.28 (Fig. 5). In the Central cluster, most admixture came from Atlantic, with *q*-values around 0.30 for two plants in a Swedish population and 0.40 for two individuals in a Norwegian population. Admixture scores for the individuals in the West cluster were always below 0.10. In the Spanish cluster, five individuals from two populations had *q*-values between 0.13 and 0.46 for the Atlantic cluster. The three *R. minor* individuals in the South cluster showed considerable admixture with Atlantic and West.

In the west, admixture with *R. angustifolius* was found in only two plants: one from DK2 and one individual from an English population, both with *q* = 0.02. This was in contrast with the eastern populations: of the nine individuals that were originally recognized as *R. angustifolius* by Vrancken et al. (2009) and now identified as belonging to the Central cluster of *R. minor*, seven had *q*-values for Central below 0.95, with considerable admixture with either Atlantic (PL1, 3 individuals) or *R. angustifolius* and/or South (BG1, BG3, RO1, 4 ind.).

In the southeastern *Rhinanthus* sp. populations, admixture was much rarer. No admixture was found in the Greek and Italian clusters. One individual in the Russian cluster had a *q*-value of 0.29 for the Greek cluster. The Italian population in the South cluster showed no admixture at all, in contrast with all the other plants in this cluster.

### Admixture with *R. minor* in *R. angustifolius*

Admixture levels varied considerably among *R. angustifolius* AFLP groups. For the main group G1, *q*-values below 0.95 were only found in three individuals (5%) in BE1, SC3, and NL5. The Romanian population RO2 (AFLP group G3) clustered with the other *R. angustifolius*, with a *q*-value of 0.11 for South in one individual. Of the 14 individuals in the original G5 cluster, six (43%) had *R. angustifolius q*-values below 0.95, and showed admixture with West, South, and Italian. As mentioned earlier, TR1 (AFLP group G3) and FR5 (AFLP group G2) had *R. angustifolius q*-values well below 0.5 and were admixtured with Russian and South (TR1) or South only (FR5).

### cpDNA haplotype sharing between *R. minor* and *R. angustifolius*

**Figure 6:**
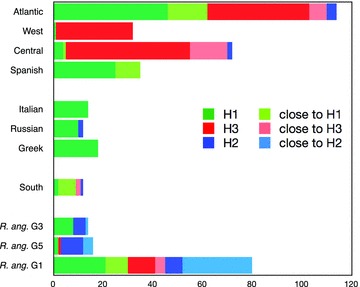
Distribution of cpDNA haplotypes over the nine AFLP clusters, with the haplotypes for *Rhinanthus angustifolius* further subdivided into the main groups detected by Vrancken et al. (2009).

**Figure 7:**
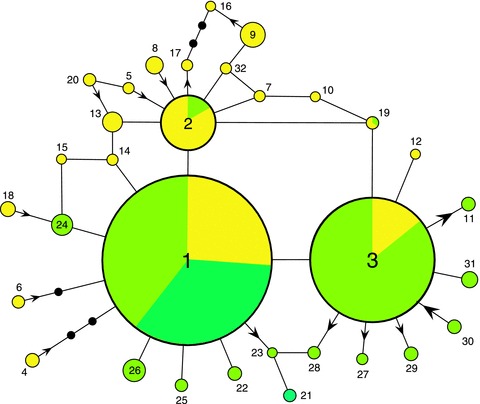
Parsimony network for all cpDNA haplotypes detected in *R. minor* s.s. (light green), *Rhinanthus* sp. (dark green), and *Rhinanthus angustifolius* (yellow).

The *R. minor* AFLP clusters differed considerably in their frequencies of the main cpDNA haplotype groups (Fig. 6). Haplotype H3 dominated in the Central and West clusters and was absent from the Spanish cluster, while H1 was dominant in the Spanish cluster, abundant in the Atlantic cluster and rare in the Central and West cluster. All three haplotypes were found in *R. angustifolius*, but H3 almost exclusively in the G1 group. The complete haplotype network (Fig. 7) shows the relative frequencies of all haplotypes found in the AFLP clusters discussed in this paper. Only haplotype H1 was shared among the three taxa, while H2, H3, and H19 occurred in both *R. angustifolius* and *R. minor*, although H2 was only found in two populations in *R. minor*, ir2 (*n* = 4) and de1 (*n* = 2).

## Discussion

### *Rhinanthus minor*: an unexpected split

The populations originally identified as *Rhinanthus minor* that were sampled in Italy, Greece, and Russia (Caucasus), although morphologically similar to this species, turned out to be strongly genetically differentiated from all other *R. minor* populations from central, western, and northern Europe. The fact that they turned out to be genetically more similar to but still distinct from *R. angustifolius* argues for treating these populations as a distinct taxon at the species level, here tentatively named *Rhinanthus* sp. At present, we only have data to show that they are closer to *R. angustifolius*, but detailed morphological examination of these populations in combination with a comprehensive phylogeny of the genus would be needed to determine the affinities of this taxon and name it appropriately. A possible candidate is *Rhinanthus personatus* (Behr.) Beg., a species described from Italy (Pignatti 1982). The Flora Europaea treats it under *R. minor* and not as a separate species, since it appears to differ morphologically from *R. minor* only in the corolla throat, which is more or less closed (von Soó and Webb 1972). Detailed morpho-logical analysis of the variation in this character both within and among the taxa *R. minor*, *R. personatus*, *Rhinanthus* sp. in Italy, Greece, and Russia is needed to establish whether the genetic divergence is reflected in a consistent morphological differentiation.

### *Rhinanthus minor*: a species phylogeography

For the *Rhinanthus minor* s.s. populations, the cpDNA and AFLP datasets showed similar patterns, suggesting that the data collected, rather than reflecting independent gene phylo-geographies, describe a reliable species phylogeography for *R. minor*. Analyses of the distribution of the genetic variation with the cpDNA and AFLP datasets showed that although some populations in the south of Europe had high genetic diversity, there was no decrease of genetic diversity with latitude in either marker type. Equal levels of variation at high latitudes is in contrast with what is usually observed for thermophilous species, which typically present the highest genetic diversity in the south and a decrease in this variation toward the north, due to repeated bottlenecks and founder effects during recolonization of previously glaciated areas (Taberlet et al. 1998).

Several hypotheses may explain the high level of genetic diversity in the north of Europe: mid-latitude survival, secondary contact in Scandinavia, hybridization with other species, notably the nordic *Rhinanthus groenlandicus*, and an eastern refugium that was not sampled at lower latitudes. We discuss each of them hereafter.

Occurring nowadays in cold regions such as Iceland and northern Scandinavia, *R. minor* may have managed to survive glacial periods under the harsh climatic conditions at the edge of the ice sheets that covered central and northern Europe during the last glaciations. This hypothesis of mid-latitude survival of *R. minor* populations is reinforced by the observation of relatively high DW values in northern areas (Table 1). High DW values indicate an abundance of rare markers, which are a proxy for genetic divergence, good indicators of historical processes, and generally associated with glacial refugia (Comps et al. 2001; Widmer and Lexer 2001; Paun et al. 2008; Winkler et al. 2010).

On the other hand, low levels of differentiation among populations, which is often observed when intermediate-latitude survival of a species is suspected (Rendell and Ennos 2002; Palmé et al. 2003), was not found among the *R. minor* populations. Instead, we see divergence along an east–west axis, with the AFLP cluster Atlantic and cpDNA haplotype H1 in the west and the Central cluster with cpDNA haplotype H3 in the east, with the West cluster in between the two other clusters in France and Belgium. The Spanish cluster was clearly differentiated from the three more northern clusters in the AFLP analysis, and also distinguished by the complete absence of haplotype H3. It is likely that the Pyrenees have formed a barrier for gene exchange both during and after the ice ages. Iceland was probably recolonized by *R. minor* in historical times by humans transporting cattle (Diamond 2005), and gene flow among the Spanish and Atlantic populations may have taken place in a similar way.

The phylogroups from central Europe extend from south to north and meet in Scandinavia. The high genetic diversity observed in Scandinavia may be caused by lineages expanding from southern regions that have come into contact in the north (Remington 1968; Taberlet et al. 1998). Still, the abundance of rare AFLP markers and the occurrence of unique cpDNA haplotypes in Scandinavia do not agree with a hypothesis of exclusively southern survival followed by colonization of the northern areas and hybridization by two distinct southern lineages. The rare markers and haplotypes found in the north should then also have been found in populations at lower latitudes, which was only the case for H27 (Norway and Austria). It is more likely that refugia were located at latitudes north of the Alps, from Belgium to the Ukraine. Unfortunately we did not sample enough populations from this region to be able to confirm that it was the direct source for the high genetic variation in the north. The Alps would contain less genetic variation today, since these would have been recolonized from the surrounding plains after the ice retreated.

A third explanation would be hybridization with another *Rhinanthus* species, notably *R. groenlandicus* (Ostenf.) Chabert, which only occurs in northern Europe above 60°N and in the mountains in Norway, plus on the Faroe Islands and Iceland (von Soó and Webb 1972). Although treated as a separate species in the Flora Europaea (von Soó and Webb 1972), *R. groenlandicus* is considered a subspecies of *R. minor* (*R. minor* ssp. *groenlandicus* [Chabert] Neuman) in Scandinavia (http://linnaeus.nrm.se/flora/), and it is possible that our samples from Norway contained genetic material from this (sub)species.

With the lack of *R. minor* samples from the east of Europe, we cannot exclude that colonization of Scandinavia occurred from a separate, more eastern lineage, but the *R. minor* samples from Poland, Bulgaria, and Romania fell within the Central cluster and did not share any haplotypes with the Scandinavian populations (Fig. 7). Furthermore, the rare cpDNA haplotypes were only found in western Scandinavia, which would not argue for an eastern origin.

In contrast to *R. minor* s.s., the data collected for the *Rhinanthus* sp. populations in Greece, Italy, and Russia did not yield useful phylogeographic patterns. The observation of the common H1 haplotype as the only one detected in Greece, Russia, and Italy together with H21, a haplotype only found in one of the Italian populations, did not give much phylogeographic information. The AFLP data did not have more helpful information for phylogeographic inferences, as each region formed an independent genetic cluster. Further sampling in Turkey and Eastern Europe would be needed to resolve the relations within this group and with *R. minor*.

### Origins of haplotype sharing

Our data confirmed the preliminary information about significant cpDNA haplotype sharing between *R. angustifolius* and *R. minor*. Although only four out of 32 haplotypes described for the two species were shared, these haplotypes were found in 83% of all *R. minor* individuals and in 60% of all *R. angustifolius* individuals. The presence of an identical cpDNA haplotype in different species may result from various and nonexclusive processes including convergent evolution, incomplete lineage sorting, and interspecific gene flow. Given the fact that hybridization between *R. minor* and *R. angustifolius*, both widespread and occurring in similar habitats, has long been shown to be successful and common where the two species meet (Campion-Bourget 1980; Kwak 1980; Ducarme and Wesselingh 2005; Ducarme et al. 2010), gene flow between these species could be one explanation for the extensive haplotype sharing. We have looked at all three hypotheses to explain our results.

Given our dataset, homoplasy is an unlikely explanation. Even if for each shared haplotype the probability of an independent identical mutation is not completely zero, since the differences between the main haplotypes consist of a single point mutation, it is hard to imagine how these independent emergences could have resulted in the predominance of these haplotypes in both species over large parts of their ranges.

Due to their position in the haplotype networks and their abundance, the shared haplotypes, especially H1, probably represent ancient haplotypes that would predate the species split and are likely to have been inherited by *R. minor*, *Rhinanthus* sp., and *R. angustifolius* from a common ancestor. This hypothesis is reinforced by the fact that the other haplotypes, which are less abundant and represent terminal haplotypes in the haplotype networks, are all species specific. Haplotype sharing due to conservation of ancestral polymorphisms has been detected in several closely related species (Charlesworth et al. 2005). In plants, this mechanism has been proposed to explain extensive allele sharing between two closely related sympatric oak species (Muir and Schlötterer 2005).

Although DNA phylogenies have been published for the Orobanchaceae (Bennett and Mathews 2006), and the Rhinanthoid clade (Těšitel et al. 2010), no complete molecular phylogeny exists for the genus *Rhinanthus*. The few molecular studies that have included *R. minor* or *R. angustifolius* (Böhme 2001; Těšitel et al. 2010) show *R. alectorolophus* as the earliest-branching lineage in the genus, based on internal transcribed spacer (ITS) sequence data. We have detected haplotypes H1, H2, H13, and H31 in *R. alectorolophus*, along with three unique haplotypes derived from H1 and H2, and *R. alectorolophus* does not hybridize with *R. minor* (R. A. Wesselingh, unpubl. data). In two other species, *Rhinanthus freynii* from Italy and *Rhinanthus rumelicus* from Greece, we found H1 and H2, respectively. This suggests that H1 and H2 are ancient haplotypes in the genus, as they are shared by several species. However, given its abundance and the number of derived haplotypes in *R. minor*, it is likely that H3 has evolved later, and only in *R. minor*. If this is indeed the case, then the presence of H3 in five *R. angustifolius* individuals across Europe can only be explained by hybridization or convergent evolution. The former explanation is very likely in population BG1, which was a mixed population with two *R. angustifolius* and two *R. minor* individuals in our dataset. The two *R. minor* plants in this population carried H3, and one of the *R. angustifolius* plants had H3 as well. The other three *R. angustifolius* populations with H3 present in one or two individuals were found in southern Sweden, the North Sea coast of the Netherlands, and in the Czech Republic, all areas where hybridization with cpDNA introgression could have occurred given the geographic distribution of *R. minor* and its haplotype H3. In none of these individuals, or in other individuals of the same populations, did we find increased levels of admixture, so if the presence of haplotype H3 is a trace of hybridization, it is likely not recent. Haplotype H12, derived from H3, was only found in a single individual in a German *R. angustifolius* population (DE2), and it may have arisen after an ancient hybridization event as well.

Haplotype H2, which is much more prevalent in *R. angustifolius*, was only found in two *R. minor* populations; one in Ireland, which was monomorphic for this haplotype, and one in southern Germany. This would suggest that H2 was not originally present in *R. minor*, but that was acquired through hybridization with *R. angustifolius*. While hybridization as a source for cpDNA haplotype introgression would be possible in Germany, *R. angustifolius* does not occur in Ireland, so hybridization in situ would be an unlikely explanation for the occurrence of H2 in an Irish population, leaving convergent evolution by a second occurrence of the single point mutation that separates H1 from H2, or an ancient polymorphism. Introgression of cpDNA from *R. angustifolius* to *R. minor* would require sufficiently extensive F_1_ hybrid formation to overcome the low germination rate of F_1_ hybrids with *R. angustifolius* cytoplasm (Kwak 1979; Campion-Bourget 1980), which makes this even more unlikely, plus the fact that in mixed populations, introgression generally takes place toward *R. angustifolius* (Ducarme et al. 2010).

### Phylogeographic consequences of gene flow

If all occurrences of H3 within *R. angustifolius* are caused by interspecific gene flow, then removing these from the cpDNA dataset might facilitate the detection of a phylogeographic signal in *R. angustifolius* (Rieseberg and Soltis 1991). However, even without H3, there is no clear geographical distribution of the two haplogroups H1 and H2 that is congruent with the division between AFLP group G1 on one side and G3 and G5 on the other. Both haplotypes H1 and H2 were probably present in both eastern and western Europe at the beginning of the expansion of *R. angustifolius*, which is likely to have coincided with an increase in human populations and livestock grazing, which creates the open grassland habitat necessary for the species. The expansion of the species into northern Europe probably predates the formation of derived haplotypes, especially from H2, which are usually restricted to one or only a few populations. While the original haplotypes were similar in the different *R. angustifolius* lineages, H3 in *R. minor* probably arose early in central Europe and spread with the Central cluster. Both species have a low natural seed and pollen dispersal, so the lack of genetic structure within G1 in *R. angustifolius*, which covers most of central and western Europe, is difficult to explain. We may hypothesize that it occurred more often in hay meadows than *R. minor*, which is more abundant on poorer, dryer soils, and that *R. angustifolius* has thus profited more from human hay and livestock transport than *R. minor*.

In conclusion, the AFLP analysis was able to unequivocally distinguish *R. minor* from *R. angustifolius*, and correctly identify hybrids between these species. Other taxa were also identified, in particular *Rhinanthus* sp. in southeastern Europe, and possibly the French population that clustered separately from *R. angustifolius*. This population formed a separate AFLP cluster, South, which also included highly divergent individuals from other populations that were on separate branches in the NJ tree, and this cluster should probably not be interpreted as a phylogenetic signal. It is possible that yet another *Rhinanthus* species is involved in the ancestry of the population from the French Pyrenees; five species are known to occur in France (Campion-Bourget 1980). To correctly identify the unknown taxa, a molecular phylogeny of the whole genus should be constructed and combined with a morphological analysis. The low resolution of traditional phylogenetic markers such as ITS in this genus (Těšitel et al. 2010) may pose problems, but our AFLP analyses have shown that sufficient genetic variation is present. Isolation-migration analysis (Becquet and Przeworski 2009) would be a powerful tool to separate incomplete lineage sorting from hybridization, but requires sufficient sequence variation, plus it assumes no hybridization with other populations than those sampled (Slotte et al. 2008), which poses problems in the case of *Rhinanthus*, where taxon limits are not clearly defined.

## Appendix A1

**Table:** *Rhinanthus angustifolius* populations studied in Vrancken et al. (2009) and rerun for AFLP comparison with *R. minor* populations

Population	Pop 2009^1^	Country	Locality	Latitude	Longitude	*n*
BE3	P1	Belgium	Doode Bemde	50°49′	4°38′	3
BG1^2^	P2	Bulgaria	Ogoja	42°54′	23°30′	4
BG2	P3	Bulgaria	Gintsi	43°03′	23°06′	4
BG3^2^	P4	Bulgaria	Dragalavtsi	42°40′	23°18′	4
CZ1	P19	Czech Republic	Bohemian forest	48°46′	13°54′	4
CZ2	P20	Czech Republic	Bílé Karpaty	48°53′	17°42′	3
DK2^2^	P5	Denmark	Arnum	55°15′	8°59′	3
FI4	P6	Finland	Joensuu	62°36′	29°45′	4
FR5	P7	France	Seissan	43°30′	0°36′	4
DE2	P11	Germany	Coerde	51°58′	7°38′	4
NL3	P12	Netherlands	Utrecht	52°06′	5°10′	4
NL4	P13	Netherlands	Meijendel	52°09′	4°23′	4
NL5	P14	Netherlands	Populierenlaantje	53°02′	6°39′	1
NL6	P15	Netherlands	Katwijk	52°12′	4°25′	3
NL7	P16	Netherlands	Wageningen	51°58′	5°40′	4
PL1^2^	P17	Poland	Bialowieza	52°40′	23°50′	4
PL2	P18	Poland	Warsaw	52°10′	21°04′	4
RO1^2^	P21	Romania	Bârsa	46°25′	22°07′	3
RO2	P22	Romania	Iasi	47°09′	27°38′	3
RU2	P23	Russia	Kovkenicy	60°38′	33°13′	4
RU3	P24	Russia	Liski	51°09′	40°00′	3
SC3	P25	Scotland	Battleby	56°27′	–3°28′	4
SE6	P26	Sweden	Ringenäs	56°41′	12°43′	4
SE7	P27	Sweden	Johanneshus	56°08′	13°42′	4
TR1	P28	Turkey	Cankiri	40°36′	33°38′	3
TR2	P29	Turkey	Hensi	39°22′	42°43′	4

1 Population numbers used in Vrancken et al. (2009).

2 Populations identified as *R. minor* (see text), the two Bulgarian populations are mixed.

## Appendix A2

Structure-sum-2011 (Ehrich 2006) summary of STRUCTURE results for the complete AFLP dataset for *Rhinanthus minor* and *Rhinanthus angustifolius*. For each value of *K*, 25 runs were performed, for which the LnP(D) values are shown individually in (a). The mean delta*K* (Evanno et al. 2005) is shown for *K* from 2 to 12 for all runs (b) and without the outlier run for *K* = 10 (c).
